# Kinetic Determination of Cytochrome *b_6_f* Activity In Vitro

**DOI:** 10.21769/BioProtoc.5677

**Published:** 2026-05-05

**Authors:** Yuval Milrad, Daniel Wegemann, Michael Hippler

**Affiliations:** 1 Institute of Plant Biology and Biotechnology, University of Münster, Münster, Germany; 2 Institute of Plant Science and Resources, Okayama University, Kurashiki, Okayama, Japan

**Keywords:** Cytochrome *b_6_f*, Plastocyanin, Photosystem I, Joliot-type spectrophotometer, Photosynthesis, Electron-flow

## Abstract

While traditional kinetic studies of the cytochrome *b_6_f* complex have frequently relied on measurements within the complex environment of intact leaves or whole-organism systems, such approaches can be limited by overlapping signals and physiological variables. This protocol advances existing frameworks by introducing a streamlined, multi-wavelength spectroscopic approach utilizing a reconstituted in vitro system to elucidate the inter-complex electron transfer kinetics between photosystem I and cytochrome *b_6_f*. Utilizing the JTS-150 pulsed spectrometer, supplied with a *Smart Lamp*, we monitored the redox transitions of P700^+^ and Cyt*f* by simultaneously measuring the absorbance changes of our isolated complexes system in six different wavelengths (546, 554, 563, 574, 705, and 740 nm). Kinetic analysis was divided into two phases: laser-induced flash kinetics and steady-state actinic induction. We resolved the second-order re-reduction of P700^+^ by plastocyanin, accounting for detector saturation constraints with a 2 ms post-flash delay. Steady-state measurements under actinic light revealed complex Cyt*f* turnover, characterized by a double-exponential decay. Furthermore, dark relaxation kinetics were used to quantify ferredoxin-mediated re-reduction of the cytochrome pool. By allowing the incorporation of specific regulatory and inhibitory factors, this methodology sets the ground for the deconvolution of competing electron pathways. It can therefore be used as a robust framework for assessing the mechanism of regulatory processes on photosynthetic flux.

Key features

• Activity measurement of isolated photosynthetic complexes.

• Assessing interactions between complexes in the photosynthetic apparatus.

## Graphical overview


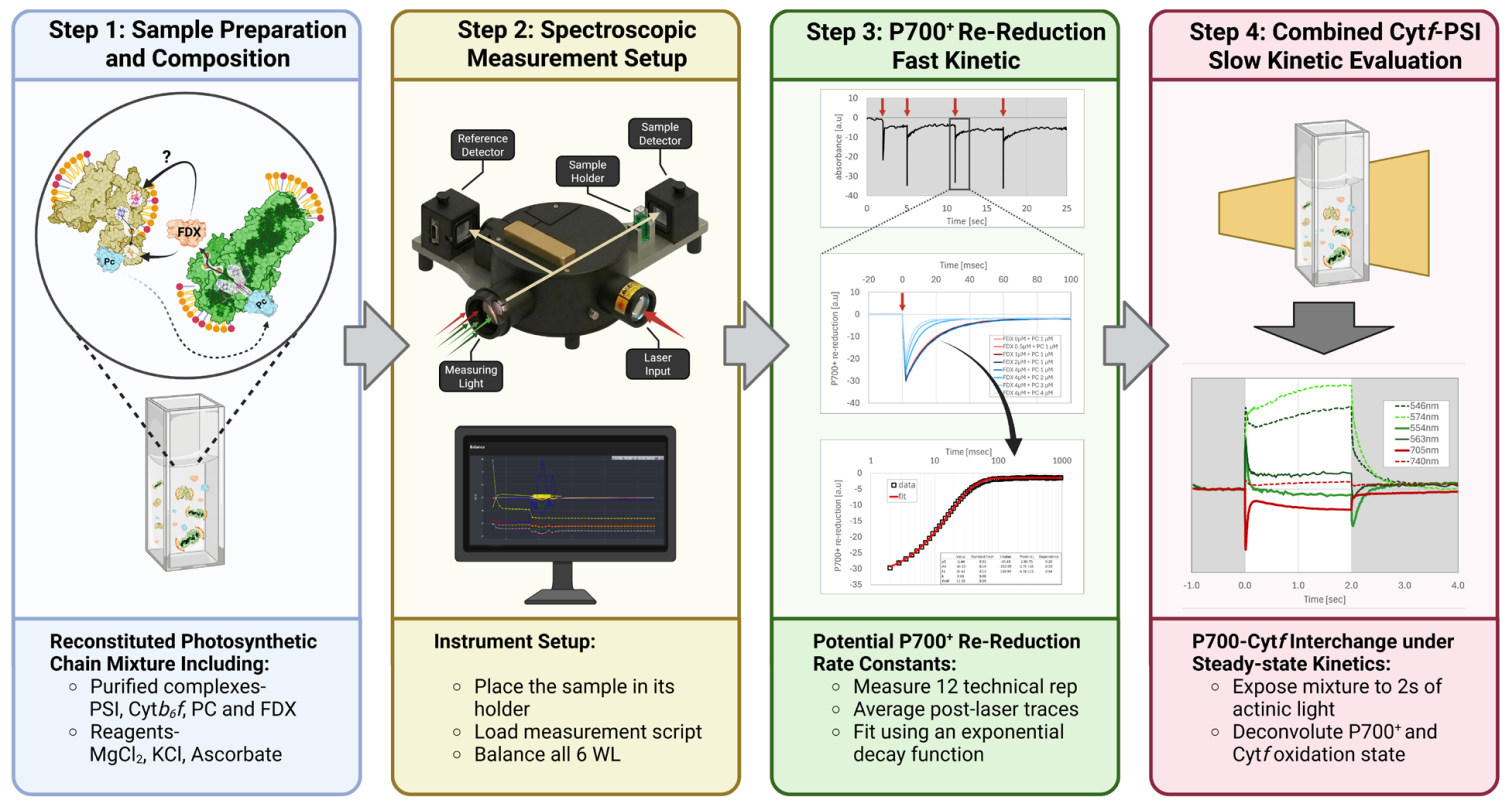


**Schematic overview of our experimental setup.** (Step 1) Reconstitution of the photosynthetic chain between cytochrome *b_6_f* (Cyt*b_6_f*, yellow) and photosystem I (PSI, green) via plastocyanin (PC, cyan) and ferredoxin (FDX, orange), using ascorbate as a reducing agent. To generate this chain, first place all purified complexes and proteins in a measuring cuvette. (Step 2) Spectroscopic measurement configuration, including sample placement, script loading, and light configuration measurement. Make sure to use the *multi-LED probing source smart lamp* and remove all filters (of both measuring and detection). Start by balancing the six wavelengths (546, 573, 554, 563, 705, and 740 nm). Make sure that no trace is saturated beyond 10 V by rotating the measuring lamp. (Step 3) Evaluation of fast P700^+^ re-reduction kinetics. Prior to each actinic light exposure, a series of three laser flashes is initiated (with an additional *laser control* pre-flash). Data from 12 technical replicates (4 measurement rotations, 3 flashes each) are averaged and fitted with an exponential decay function to determine rate constants, amplitudes, and other parameters. (Step 4) Analysis of slow kinetics under steady-state conditions. The mixture is exposed to 2 s of actinic light to assess the kinetic relationship between the oxidative state of the two complexes (PSI-*Cytb_6_f*). Use all 6 wavelengths to deconvolute P700^+^ and Cyt*f* state. Add increasing FDX and PC concentrations (for additional suggestions, see Procedure. Additional Perspectives and Future Applications) to assess their impact on kinetics.

## Background

**Figure 1.**
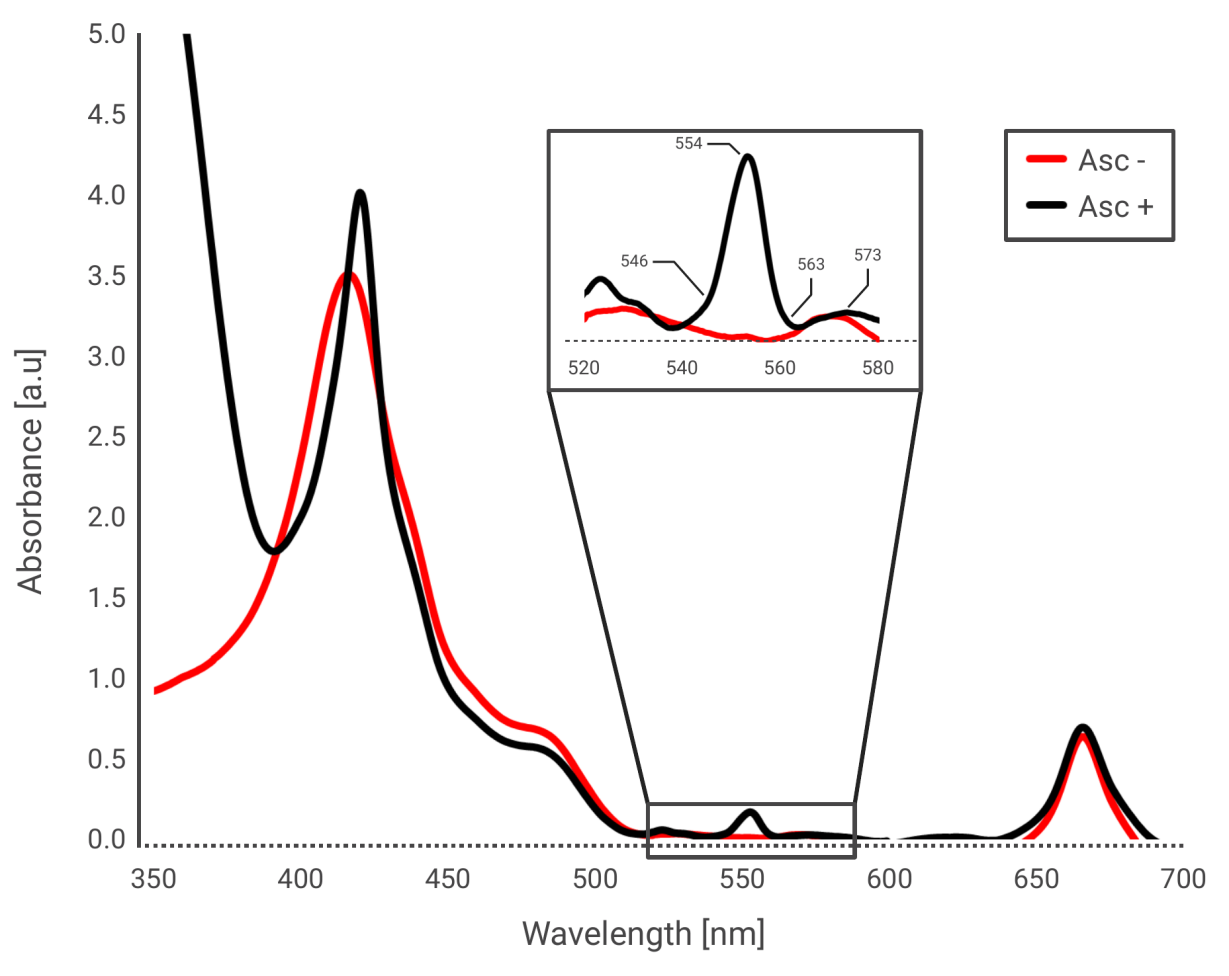
Absorbance spectrum of isolated Cyt*b_6_f*. Isolated complexes of Cyt*b_6_f* were diluted in Cyt*b_6_f* purification solution (CPs, see Recipe 8). The complexes were tested in the absence (red) or presence (black) of 10 mM ascorbate, a known reducer. Insert focuses on the region between 520 and 580 nm. The absorbance increases dramatically and peaks at 554 nm, due to the presence of *c*-type hemes, such as in the subunit of Cyt*f.*

The cytochrome *b_6_f* (Cyt*b_6_f*) complex serves as the essential electronic bridge within the photosynthetic electron transport chain [1]. Situated between the two photosystems, it mediates electron transfer from the mobile, membrane-bound carrier plastoquinol (PQH_2_) to the luminal shuttle plastocyanin (PC); the latter then delivers the electrons to photosystem I (PSI) (Graphical overview). This process is coupled with the translocation of protons across the thylakoid membrane, contributing significantly to the proton motive force (PMF) required for ATP synthesis [2,3]. Since the oxidation of PQH_2_ at the Qo site of Cyt*b_6_f* is considered to be a major rate-limiting step, the complex acts as the primary gatekeeper of photosynthetic flux, directly influencing photosynthesizers’ ability to balance energy assimilation with photoprotection [4,5]. The structural and spectral complexity of the Cyt*b_6_f* complex is defined by its chromophores, centered around four internal heme groups, each of which possesses unique spectroscopic properties. These include two b-type hemes, *b_l_* and *b_h_*, located on the cytochrome *b_6_* subunit, and two c-type hemes: *c_i_* (also known as *c_n_*), located on the stromal side of Cyt*b_6_*, and heme *f*, located on Cyt*f*, serving as the electron donor site for PC [6,7]. Because each of these hemes exists in a slightly different protein environment, their absorbance spectra shift relative to one another. This spectral diversity enables researchers to dissect the activity of the complex with remarkable precision. By choosing specific wavelengths, one can isolate the electronic transitions of a single heme amidst the background of the others [8]. Most studies monitored the Cyt*f* reduction state. In its reduced state, Cyt*f* exhibits a sharp, prominent absorption peak at 554 nm, known as the α-band (**Figure 1**) [9]. This peak is highly sensitive to the redox state of the iron center; upon oxidation (by PC, in our case), the absorbance at 554 nm decreases sharply. This relatively high extinction coefficient allows for the detection of minute changes in electron occupancy even in complex biological samples like intact leaves or isolated thylakoid membranes. In addition to this, the ability to monitor the b-hemes (typically around 560 nm) allows for the study of the Q-cycle, a bifurcation mechanism by which the complex doubles its proton-pumping efficiency. Most contemporary research takes advantage of these overlapping but distinct fingerprints to map the internal electron pathway of the complex.

Spectroscopic analysis of cytochrome *b_6_f* kinetics was fundamentally established by Joliot and Joliot, who introduced the pulsed LED-based spectrophotometry required to resolve rapid absorbance changes in photosynthetic complexes [10]. While later work refined these techniques to quantify absolute electron fluxes in intact systems [11], these approaches are often complicated by the physiological variables of whole organisms. Our protocol builds upon these foundational kinetic principles by applying them to a reconstituted in vitro system using purified complexes from green algae and other organisms. This allows for a streamlined, high-resolution analysis of inter-complex electron transfer without the interference of overlapping signals inherent to more complex biological matrices. To capture these transitions in real-time, the photosynthetic community has turned to Joliot-type spectroscopy (JTS) as a well-established method [12–14]. This type of spectral measurement relies on a series of weak monochromatic light pulses that are sent through the sample at microsecond intervals. By measuring the transmission of these probe pulses, we can reconstruct the kinetic trace of Cyt*f* oxidation and its subsequent re-reduction. This grants it the ability to provide millisecond-scale temporal resolution, while maintaining high sensitivity to these specific heme absorbance changes. While recent advancements have successfully applied JTS to monitor Cyt*b_6_f* kinetics in vivo [8,15], there remains a significant need for a streamlined, accessible in vitro system within the photosynthetic community. In vivo measurements, though physiologically relevant, are often confounded by the sheer complexity of the cellular environment and competing electron sinks. Conversely, traditional in vitro studies focusing on Cyt*b_6_f* interactions have historically relied on ultra-fast techniques such as stopped-flow spectroscopy [9,16]. While powerful, stopped-flow systems are often limited to tracking a single, isolated electron transfer event and lack the flexibility to integrate multiple regulatory components. Our protocol addresses this gap by creating a modular, reconstituted system that incorporates isolated PSI complexes to act as the primary oxidizer. In this setup, light exposure triggers PSI to oxidize PC, which subsequently oxidizes Cyt*f*, allowing us to monitor the entire kinetic chain at a resolution previously reserved for much more complex setups. By using PSI as a light-mediated biochemical switch, we can move beyond these limitations and begin adding specific regulatory factors, such as ferredoxin (FDX), into the reaction mix. This allows us to investigate multifaceted interactions, including the potential role of FDX in reducing the cytochrome complex from the stromal side—an area of ongoing investigation that this protocol is uniquely equipped to explore. One other advantage of using JTS for this type of research is the ability to incorporate a high-intensity laser flash, which synchronizes the photosynthetic population. This flash, typically lasting only a few nanoseconds, induces a single turnover of PSI, leading to the immediate oxidation of the P700 reaction center. Since we change the concentration of electron carriers such as PC, we de facto alter the kinetics and possibly reach the maximal capacity of PSI, which operates as an oxidizer for our system. Therefore, tracking the rate at which the P700 reaction centers are being re-reduced by the electron carrier grants us the ability to monitor the system. In addition, since we are not really limited by the number of simultaneously measured wavelengths, we can track the slow kinetics of PSI oxidation in light and its re-reduction in darkness. One of the key concepts guiding us in this type of research is attempting to evaluate the effect of Cyt*b_6_f* on the apparent concentration of PC, which is available to PSI. Furthermore, since the kinetics of P700^+^ re-reduction serves as a mirror view of Cyt*f* oxidation, the technique described herein may prove beneficial in future studies.

## Materials and reagents

**Biological materials**

1. The purified complexes used in this study (PSI, Cyt*b_6_f*, PC, and FDX) were isolated from green algae following the specific protocols described in [17]. These procedures were followed exactly as described without any further modifications. Each protein fraction was verified for purity and activity via absorbance spectroscopy and SDS-PAGE prior to being aliquoted and stored at -80 °C.

**Reagents**

1. 3-(Morpholin-4-yl)propane-1-sulfonic acid (MOPS) (Sigma, catalog number: 106129)

2. Antimycin A (AA) (Sigma, catalog number: A8674)

3. Ascorbate (Asc) (Sigma, catalog number: 255564)

4. Hydrochloridic acid (HCl) (Roth, catalog number: 7647-01-0)

5. Magnesium chloride (MgCl_2_) (Sigma, catalog number: M8266)

6. Potassium chloride (KCl) (Sigma, catalog number: P9541)

7. Trans-PCC α-maltoside (t-PCCαM) (Glycon, catalog number: D99019)

8. Tricine (C_6_H_13_NO_5_) (AppliChem, catalog number: A10585)

9. Water (ddH_2_O), in-house Millipore-purified water (18.2 MΩ/cm)

**Solutions**

1. MOPS stock, pH 7.0, 1 M (see Recipes)

2. Tricine stock, pH 7.8, 1 M (see Recipes)

3. KCl stock, 3 M (see Recipes)

4. Ascorbate stock, 1 M (see Recipes)

5. MgCl_2_ stock, 1 M (see Recipes)

6. t-PCCαM stock, 10% (see Recipes)

7. JTS activity solution (JAs) (see Recipes)

8. Cyt*b_6_f* purification solution (CPs) (see Recipes)

9. PSI purification solution (PPs) (see Recipes)

10. Plastocyanin column buffer (PC-B) (see Recipes)

**Recipes**

**1. MOPS stock, pH 7.0, 1 M**

**Table:** 

Reagent	Final concentration	Quantity or Volume
MOPS	1 M	20.9 g
ddH_2_O	n/a	Top up to 100 mL

a. Set pH to 7.0 using HCl 5 M.

b. Keep cool (4 °C) to avoid contaminations; life span ~6 months.

**2. Tricine stock, pH 7.8, 1 M**

**Table:** 

Reagent	Final concentration	Quantity or volume
Tricine	1 M	17.9 g
ddH_2_O	n/a	Top up to 100 mL

a. Set pH to 7.8 using HCl 5 M.

b. Keep cool (4 °C) to avoid contaminations; life span ~6 months.

**3. KCl stock, 3 M**

**Table:** 

Reagent	Final concentration	Quantity or volume
KCl	3 M	224 g
ddH_2_O	n/a	Top up to 100 mL

Keep at room temperature (24 °C); life span unlimited.

**4. Ascorbate stock, 1 M**

**Table:** 

Reagent	Final concentration	Quantity or volume
Ascorbate	1 M	198 mg
ddH_2_O	n/a	Top up to 1 mL

Keep in a dark tube. It is better to use a fresh solution mixed at the day of experimenting; short lifespan.

**5. MgCl_2_ stock, 1 M**

**Table:** 

Reagent	Final concentration	Quantity or volume
MgCl_2_	1 M	2.38 g
ddH_2_O	n/a	Top up to 100 mL

Keep cool (4 °C) to avoid contamination; life span unlimited.

**6. t-PCCαM stock, 10%**

**Table:** 

Reagent	Final concentration	Quantity or volume
t-PCCαM	10%	1 g
ddH_2_O	n/a	Top up to 10 mL

Dissolves better in hot water. Aliquot to 1 mL tubes and keep at -20 °C to increase lifespan (~6 months).

**7. JTS activity solution (JAs)**

**Table:** 

Reagent	Final concentration	Quantity or volume
MOPS (Recipe 1)	30 mM	35 mL
t-PCCαM (Recipe 6)	0.01%	1 mL
ddH_2_O	n/a	Top up to 1 L

Keep cool (4 °C) to avoid contaminations; life span 2–3 months.

**8. Cyt*b_6_f* purification solution (CPs)**

**Table:** 

Reagent	Final concentration	Quantity or volume
JAs (Recipe 7)	MOPS 30 mM t-PCCαM 0.01%	100 mL
KCl (Recipe 3)	30 mM	1 mL

Keep cool (4 °C) to avoid contaminations; life span 2–3 months.

**9. PSI purification solution (PPs)**

**Table:** 

Reagent	Final concentration	Quantity or volume
JAs (Recipe 7)	MOPS 30 mM t-PCCαM 0.01%	100 mL
MgCl_2_ 1 M (Recipe 5)	5 mM	500 μL

Keep cool (4°C) to avoid contaminations; life span 2–3 months.

**10. Plastocyanin column buffer (PC-B)**

**Table:** 

Reagent	Final concentration	Quantity or volume
Tricine 1 M (Recipe 2)	25 mM	25 mL
KCl 3 M (Recipe 3)	10 mM	3.3 mL
dH_2_O	n/a	Top up to 1 L

Keep cool (4 °C) to avoid contaminations; life span ~6 months.

## Equipment

1. Joliot type spectrophotometer -150 (Bio-Logic) designed for fast kinetics and simultaneous multi-wavelength characterization.

a. Smart lamp (detection): multi-LED probing source with integrated interference filters; 546, 554, 563, 574, 705, and 740 nm wavelengths used (standard kit for cytochrome *b_6_f* and P700); pulsed LED measurements can be acquired as fast as 10 μs between samples.

b. Actinic light source, type: dual-ring LED array (integrated into the optical bench); output: 630 nm (red) and 720 nm (far-red); intensity range: 0 to >5,000 μE/m^2^/s^1^.

c. Detection system: Detectors: two large-area high-speed Si PIN photodiodes (signal and reference); resolution: 18-bit ADC for high dynamic range; sensitivity: capable of resolving absorbance changes down to 10 × 10^-5^ OD.

d. Synchronization: Integrated BNC ports for microsecond-precision triggering of the Nd:YAG laser and external flash lamps.

2. Laser flash (Quantel, Lumibird Group), Nd:YAG (neodymium-doped yttrium aluminum garnet) solid-state laser; primary output: frequency-doubled to 532 nm (green); wavelength shifting: the 532 nm beam is used to pump a red dye filter/cavity (e.g., using LDS 698 dye); effective output wavelength: saturating red flash cantered at ~640–700 nm; pulse duration: ultra-short pulses of ~6–10 ns; pulse energy (strength): calibrated to ~20–30 mJ at the source.

*Experimental rationale: The energy is adjusted to ensure a single saturating turnover of all photosystem I (PSI) reaction centers within the cuvette while the red shift prevents spectral interference with the JTS detection wavelengths.*

## Procedure

**A. Sample preparation and composition**

1. Perform assays in a 1 mL quartz cuvette with a final working volume of 400 μL in JAs buffer, supplemented with ascorbate 10 mM, MgCl_2_ 2.5 mM, and KCl 7.5 mM. The basal reaction mixture contains isolated PSI (250 nM, calibrated per reaction center), isolated Cyt*b_6_f* (2.13 μM), and PC (recombinant) (1 μM).

*Notes:*

*1. For each biological replicate, a control measurement was performed in the absence of Cyt*b_6_f. *This served to isolate the P700 signature and confirm that the green region absorbance changes were specifically attributable to the cytochrome complex.*

*2. The concentration of photosystem I (PSI) was determined via chlorophyll quantification in 80% acetone. To ensure accurate stoichiometry within the reconstituted system, the PSI concentration was adjusted to your target concentration, e.g., 50 nM, of reaction centers (RC), based on the established ratio of 217 Chl per PSI RC for* Chlamydomonas reinhardtii. *This calibration allows for the precise determination of electron transfer rates per functional unit.*

2. To elucidate the inter-complex electron transfer kinetics, add plastocyanin (PC) and ferredoxin (FDX) sequentially at the following concentrations: FDX titration, 0.5, 1, 2, and 4 μM; PC titration: 2, 3, 4, and 5 μM.

**B. Spectroscopic setup (JTS-150 configuration)**

1. To simultaneously conduct all detections, use the Biologic "Smart Lamp" LED system, allowing for rapid automated switching between discrete wavelengths.

2. Monitor absorbance changes at 546, 554, 563, 573, 705, and 740 nm.

*Notes:*

*1. 554 nm and 563 nm target Cytf and Cytb, respectively, while 705 nm monitors P700 redox states.*

*2. 546/573 nm serve as isosbestic/reference points for Cytf and Cytb, while 740 nm serves as a reference for P700.*

3. To enable simultaneous measuring of both the green (540–580 nm) and far-red (700^+^ nm) regions, remove all physical filters from the detector path.

*Note: Although orange actinic light (350 μE/m^2^/s^1^) was present, the JTS-150 detectors utilize pulsed-probe technology (see Biologic product page for further details). Since the detector only integrates the signal during a specified measurement window, which is synchronized with the flashes of the measuring “Smart Lamp,” the continuous background actinic light is treated as a constant offset and subtracted, preventing sensor saturation or interference with kinetic data*.

4. At the beginning of the initial measurement, you are required to balance the detector signal. Here, set the balance to “Automatic” and set all detection values to 5 V.

*Note: To improve signal/noise ratio, twist the smart lamp to facilitate an increased signal voltage for the reference measurements of 554 nm and (if applicable) 705 nm.*

**Critical:** When balancing, make sure that no reference detection surpasses the maximal voltage (10 V).

**C. Measurement procedure and sequence architecture**

**Figure 2.**
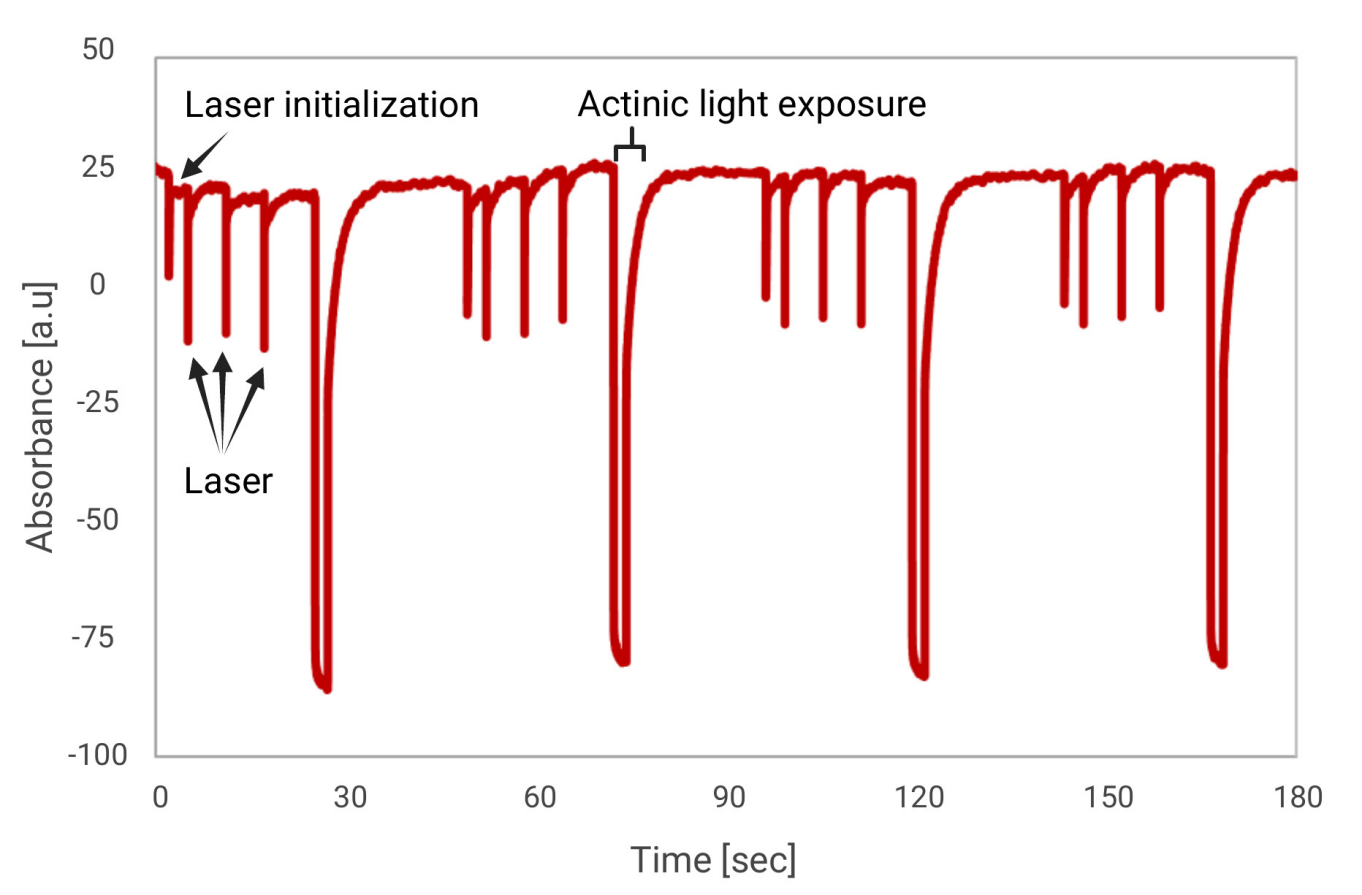
Exemplary trace. The measurement includes four technical replicates. Each begins with a probe-laser flash to initialize laser activation, followed by three laser flashes. Flowing a relaxation period, actinic light (orange ring, 350 μE/m^2^/s^1^) is activated for 2 s, followed by 20 s of darkness. To simplify, we present here only the measured absorbance at 705 nm for a sample containing the experimental initial setup (including Cyt*b_6_f* and PSI), accompanied by FDX (4 μM) and PC (4 μM).

*Note: Each measurement consisted of four technical replicates per test to ensure statistical robustness and decrease signal/noise ratio. The sequence is structured into two distinct phases: laser-induced flash kinetics and steady-state actinic flux, followed by dark relaxation (* ***Figure 2***).

1. To resolve the rapid electron transfer between Cyt*b_6_f* and P700, apply a series of four laser flashes.

a. Use the first flash to verify laser stabilization and adequate signal.

b. Implement an initial delay of 700 μs to exceed the measurement resolution. A critical delay of 2 ms must be maintained between the laser flash and the first probe pulse.

*Note: The 2 ms delay is strictly dictated by the mechanical constraints of the laser shutter and light diffraction within the cuvette. In the absence of specialized P700 interference filters (removed to allow multi-wavelength green/red detection), shorter delays resulted in diffracted laser light saturating the detector. The initial detection can vary between systems.*

2. Following the laser phase, subject the sample to a dark pause to reset the redox states before driving the full inter-complex chain.

a. Dark pause: 2 s.

b. Actinic phase: 2 s of continuous actinic light to reach steady-state turnover.

c. Recovery phase: 20 s of detection in darkness to monitor the re-reduction of P700 and the oxidation/reduction cycles of the Cyt*b_6_f* pool.

3. Use the following sequence command:

20(50msD)T4(10(200msD)A[on]10usA[off]140usB[on]10usB[off]2000usD10(200msD)3(20(50msD)A[on]10usA[off]140usB[on]10usB[off]1140us{700us,100,5s,D})10(200msD)20(50msD){1200us,100,2s, H350usD50usG[8000uE]}H{1200us,200,20s,D})

This sequence can be summarized into the segments shown in Table 1.

**Table 1.** Sequence breakdown segmentation

Segment	Function	Sequence
Sequence initiation	Dark baseline established via probe pulses; establish the number for technical replications	20(50msD) T 4(
Laser initialization	Validate laser operation and signal stability	10(200msD) A[on]10usA[off]140usB[on]10usB[off]2000usD10(200msD)
Laser pulse	High-energy excitation for P700 oxidation	3(20(50msD) A[on]10usA[off]140usB[on]10usB[off]1140us
Post-laser detection	Detection of P700 re-reduction	{700us,100,5s,D})
Pre-actinic light delay	Mitigation of diffraction and detector saturation	10(200msD)20(50msD)
Actinic light	Monitoring Cyt*f* oxidation and P700 steady-state activity	{1200us,100,2s,H350usD50usG[8000uE]}H
Dark recovery	Monitoring cytb6f re-reduction rates	{1200us,200,20s,D})

**D. Data analysis and signal processing**

Data acquisition is performed using the JTS-150 proprietary software (Bio-Logic). However, while the provided software is capable of performing all necessary deconvolutions and kinetic calculations (and users are advised to refer to the manufacturer’s manual for these standard procedures), the calculations in this protocol were performed manually in Microsoft Excel using the formulas listed below. This approach was taken to expand the analytical view and to validate that the resulting traces were consistent with the original protocol and established spectral coefficients. Data analysis and curve fitting (kinetic deconvolution and rate constant determinations) were performed using Origin.Pro 2024 (OriginLab Corporation, Northampton, MA, USA). To mimic the same analysis, follow the following steps:

1. Spectral deconvolution and baseline correction: To isolate the signals for Cyt*f* and Cyt*b*, perform a linear baseline correction using two reference wavelengths (546 nm and 573 nm) where cytochrome absorbance is not dependent on its reduction state.

a. Initial Cyt*f* correction: Since there is some overlap between the absorbance of the heme groups, to eliminate the effects of Cyt*b* on the calculated Cyt*f* trance, first correct the trace by subtracting 27% of the measured detection for 563 nm, as discussed in [18], by using the following formula:

$$D_{554}^{*}=D_{546}-0.27\times \left(D_{563}-D_{546}\right)$$

b. Signal correction: For every detection point, calculate a linear slope between the optical densities (D) at 546 and 573 nm. The corrected baseline value (D_corr_) for a target wavelength is determined as follows:

$${WL}_{corr}=WL\times \left(\frac{D_{546}-D_{573}}{546-573}\right)+\left(D_{546}-546\times \left(\frac{D_{546}-D_{573}}{546-573}\right)\right)$$

Where D are the detections at a given wavelength, and WL could be either 554 for Cyt*f* or 563 for Cyt*b*.

c. Signal isolation: The specific redox signal can then be calculated as:

Cytf = $D_{554}^{*}$ – 554_corr_

Cytb = D_563_ – 563_corr_

P700 Kinetics: The P700 signal is isolated by a simple differential subtraction, as follows:

P700 = D_705_ – D_740_

**Figure 3.**
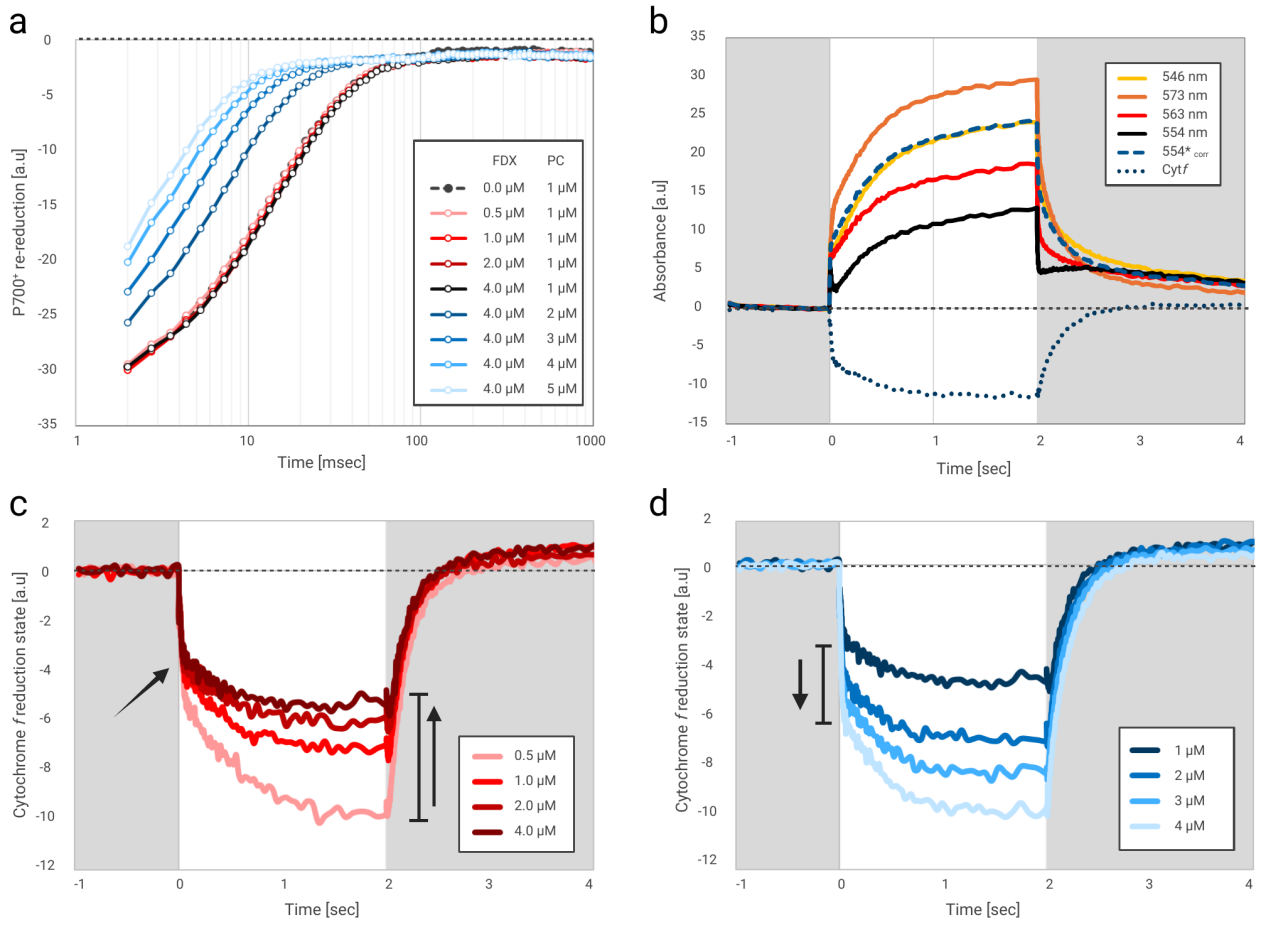
Assembled kinetic data. (a) Absorbance changes of isolated photosystem I complexes, following a laser flash, were detected for 5 s (here, the first second is presented). The result shows the averaged data of 12 technical replicates per trace. Tests examined the re-reduction rate with increasing FDX concentrations (red gradient), which showed no change. Following that, an increasing concentration of PC was added to the tested mixture (blue gradient). The effect of actinic light (orange ring, 350 μE/m^2^/s^1^) was examined then. (b) Four green wavelengths were detected at 546 nm (yellow), 573 nm (orange), 563 nm (red), and 554 nm (black). Signal correction was applied for Cyt*f* reduction kinetic determination (dashed blue), and the trace for Cyt*f* oxidation/reduction was calculated (dotted blue). Such calculations were conducted with all previously specified tests. (c) Increasing FDX concentrations (red gradient) showed no changes in the first-order kinetics (see arrow) and diminished steady-state oxidation of Cyt*f* (following 2 s of illumination). (d) On the other hand, increasing concentrations of PC increase the first-order kinetics rate, which results in an increased steady-state oxidation.

The resulting traces and the correction methodology are illustrated in **Figure 3**.

2. Generate an average of 12 measurements (3 data flashes per 4 technical replicates) to enhance the signal/noise ratio Cyt*f*, Cyt*b*, and P700 (**Figure 3**). Following the laser flash, P700^+^ re-reduction typically displays two phases:

a. First-order phase: Represents electron transfer from PC already pre-bound to PSI in a complex before the flash. With a half-life of approximately 4 μs [19], this phase is below our detection resolution, due to the imposed delay (2 ms).

*Note: We can assess it by looking at the initial amplitude of the trace; since the first detection is past the initiation of the second phase, it will be composed of a mixture of the two.*

b. Second-order phase: Represents the binding of reduced, free-diffusing PC to PSI. The second-order rate is sensitive to the kinetic properties and concentrations of both PSI and PC [17].

*Note: A high affinity between PC and Cytb_6_f may decrease the apparent concentration of free PC available for PSI reduction, slowing the observed rate.*

3. Data from 4 technical replicates are averaged. The analysis focuses on the window starting 1 s before AL, the 2-s AL period, and 2 s of subsequent dark relaxation (**Figure 3**).

AL Phase (Steady State): Cyt*f* and P700 kinetics are fitted using a double exponential decay.

a. Fast phase: t_1/2_ ~10 ms, reflects the initial rapid turnover.

b. Slow phase: t_1/2_ ~500 ms, reflects the transition toward a steady-state equilibrium.

c. Dark phase (post-AL): Upon turning off the light, Cyt*f* re-reduction follows a single exponential decay. This rate is attributed to ferredoxin (FDX)-mediated re-reduction.

**E. Additional perspectives and future applications**

Mechanism for ferredoxin- cytochrome b_6_f reduction: A key uncertainty remains on whether FDX reduces Cyt*f* directly or via the Cyt*b* hemes. In general, the redox potential of FDX is high enough to reduce all heme groups, and the specific site where it interacts with Cyt*b_6_f* in this isolated system is not clear (as discussed in [17]). Further research is required on the subject, and we urge the scientific community to pursue this topic. Our proposed electron flow scheme, including the potential bypasses or direct reduction pathways, is shown in the **Graphical overview**.

The reduction state of Cyt*b* is generally studied via spectroscopic evaluation of the absorbance changes at 563 nm. However, here we encounter two issues:

a. The absorbance changes of Cyt*b* are not as prominent as Cyt*f* since, in this experimental setup, the primary electron donor for Cyt*b* (i.e., plastoquinol) is absent.

b. There is a grave overlap between Cyt*f* and Cyt*b* absorbance change, which could technically overshadow differences observed at 563 nm.

For these reasons, at this point, we cannot undisputedly claim that the observed absorbance changes can reflect the reduction state of Cyt*b*. Further insight into the matter would require additional investigations, which we hope to cover in future studies.

This protocol provides a template for exploring further interactions within the photosynthetic chain. Future studies using this setup may include:

a. FNR interactions: Testing the impact of Ferredoxin-NADP^+^ Oxidoreductase on the re-reduction kinetics., as was proposed by [20,21].

b. Regulatory proteins: Assessing how auxiliary proteins such as STT7 [22], pgr5 [23], or PetP analogs [24] influence the Cyt*b_6_f* complex under different physiological states.

c. Environmental interface studies: Testing the effects of environmental conditions (pH, salinity, etc.) on complex formation between Cyt*b_6_f* and PC.

d. Inhibitor studies: Characterizing blockades on Cyt*b_6_f* activity using specific inhibitors such as:

i. Antimycin A: postulated to target the Qi site on the stromal side [18].

ii. DBMIB: blocking the Rieske FeS to Cyt*f* transfer) [25].

iii. DNP-INT: inhibits PQH_2_ oxidation at the Qo site [25].

## Validation of protocol

This protocol or parts of it has been used and validated in the following research articles:

- Milrad et al. [17]. Insights into plastocyanin–cytochrome b6f complex formation: The role of plastocyanin phosphorylation. *Plant Physiol*.

## General notes and troubleshooting

We list here the potential issues that may arise from using slightly different instrumentation or minor deviations from the protocol.

**Table:** 

Step	Potential problem	Possible cause	Recommendation
Step C1a	Low/inconsistent P700^+^ signal	Laser misalignment or beam divergence after the red dye cavity.	Use a fluorescent alignment card at the cuvette holder to ensure the red beam is centered.
Step C1a	Weak saturating flash	Photobleaching/degradation of the LDS 698 dye.	Replace the dye in the filter cavity if the output color shifts or energy drops below ~20 mJ.
Step C1b	Signal clipping or saturation	LED measuring pulse or laser intensity is too high for the detector gain	Decrease the smart-lamp intensity or increase the "blind spot" window to 2 ms.
Step C2	Rapid signal decay/drift	Anaerobic conditions or mediator (ascorbate) exhaustion.	Ensure the cuvette is properly sealed or refresh the mediators if measurements exceed 30 min.
